# A Research on Functional Status, Environmental Conditions, and Risk of Falls in Dementia

**DOI:** 10.1155/2014/769062

**Published:** 2014-05-19

**Authors:** Sima Ataollahi Eshkoor, Tengku Aizan Hamid, Siti Sa'adiah Hassan Nudin, Chan Yoke Mun

**Affiliations:** ^1^Institute of Gerontology, Universiti Putra Malaysia, 43400 Selangor, Malaysia; ^2^Institute for Behavioral Research, 50590 Kuala Lumpur, Malaysia

## Abstract

This study aimed to determine the effects of disability, physical activity, and functional status as well as environmental conditions on the risk of falls among the elderly with dementia after adjusting for sociodemographic factors. Data were derived from a group including 1210 Malaysian elderly who were demented and noninstitutionalized. The study was a national cross-sectional survey that was entitled “Determinants of Health Status among Older Malaysians.” Approximately 17% of subjects experienced falls. The results showed that ethnic non-Malay (OR = 1.73) and functional decline (OR = 1.67) significantly increased the risk of falls in samples (*P* < 0.05). The findings indicated that increased environmental quality (OR = 0.64) significantly decreased the risk of falls (*P* < 0.05). Disability, age, marital status, educational level, sex differences, and physical activity were found irrelevant to the likelihood of falls in subjects (*P* > 0.05). It was concluded that functional decline and ethnic non-Malay increased the risk of falls but the increased environmental quality reduced falls.

## 1. Introduction

Dementia increases the risk of falls among the elderly by the impairment of judgment, gait, and visual-spatial perception as well as the ability to recognize and avoid hazards [1, 2]. Factors such as disability [3, 4], physical activity [5], functional status [6], cognitive function [7], and the environmental conditions [7–12] can influence the risk of falls in the elderly. The environment including external conditions such as warmth, noise, and light surrounding people [12] can increase the rate of falls in later life [11]. Dementia aggravates age related changes in sensory receptions in the body, which can increase the sensitivity for environmental conditions [10], and the risk of falls consequently.

Disability defined as difficulty to perform daily activities [4, 13] can cause limitations in body functioning and structures and consequently inhibit activities and participations in particular environmental settings [10]. Performance of daily living activities is associated with balance in the rest or moving from one position to another [14]. Limited activities can increase the risk of falls two to four times [4].

On the other hand, physical activities positively affect health and cognitive function in the elderly. Regular exercises improve muscle strength, reaction time, balance, and gait [15], which help to prevent and reduce developing secondary conditions caused by functional decline and physical diseases [16]. However, this study is proposed to assess the effects of disability, physical activity, functional status, and environmental conditions as well as sociodemographic factors on the likelihood of falls in the elderly with dementia.

## 2. Methods

The project was registered in the National Medical Research Register (Project Code: NMRR-09-443-4148). Approval and permission for conducting the study were received from the Ethical Committee of the Ministry of Health. It was a national cross-sectional survey titled “Determinants of Health Status among Older Malaysians” and carried out in cooperation with the Institute for Health Behavioral Research, the National Institute of Health, the Ministry of Health, and the Institute of Gerontology, Universiti Putra Malaysia (UPM). The research included 1210 elderly with dementia who were Malaysian population aged 60 years and above living in noninstitutional places. The elderly residing in institutions and bedridden were excluded and were not recruited into the study. The data were collected by trained interviewers and the average duration of interview lasted approximately 60 minutes. Samples represented the Malaysian population in terms of age and were collected from Peninsular Malaysia that was divided into four zones of North, South, West, and Central. The current study determined the effects of age, ethnicity, disability, marital status, physical activity, sex differences, educational level, environmental condition, and functional status on the risk of falls.

In this study, minimental score less than 26 was reported to be demented score using the minimental state examination (MMSE) [17]. In addition, the definition of falls was according to the International Classification of Diseases (ICD-9) [18], which excluded falls due to the loss of consciousness, epileptic seizure, and sudden onset of paralysis such as stroke. History of falls was referred to as having at least one fall, at day or night time, during the past six months. Furthermore, the instrument of short form IPAQ was used to measure physical activity [19].

The environmental settings were evaluated based on qualities and facilities found in the living places of subjects [10, 20]. The condition of environment was related to strength, lighting, ventilation, noise, cleanliness, sanitation, electrical supply, water supply, and toilet types in places. Functional status was measured by Get Up and Go Test (GUGT) [21, 22], which was based on the ability to rise from a chair, walk three meters, turn, and return to the chair. Functional decline was considered when the time lapsed more than 20 seconds [22]. The measurement of disability was by the schedule of 12-item WHO-DAS II [23] classified from the International Classification of Functioning (ICF), Disability and Health. The schedule marked 60 and included 12-item and 6 domains. The subjects with more than 50% impairment were considered as having disability.

### 2.1. Statistical Analysis

The prevalence of falls was calculated for whole samples in regard to their age, ethnicity, disability, marital status, physical activity, educational level, sex differences, functional status, and environmental conditions. Bivariate analysis was applied using a series of chi square tests to examine the association of each variable with the risk of falls in the elderly with dementia. The multiple logistic regression analysis was used to identify the effects of age, ethnicity, disability, sex differences, marital status, educational level, environmental conditions, functional status, and physical activity on the risk of falls in respondents. Odds ratios (OR) with 95% confidence intervals (95% CI) were computed. The critical level for rejection of the null hypothesis was considered to be a *P* value of 5%, two-tailed. All analyses were performed using the Statistical Package for the IBM Social Sciences (SPSS) software version 20.0 (Chicago, IL, USA).

## 3. Results

Analysis was carried out on data from 1210 respondents who were the Malaysian elderly with dementia. The prevalence of falls among respondents was 17% (95% CI: 15.01–19.24) (Table 1). The results showed that the risk of falls in the environments with lower quality (23.6%) was higher than that in the environments with higher quality (15.1%). Furthermore, the percentage of falls was as high as 12.7% for samples who live in the environment with higher facility and 17.7% for those in the environment with lower facility. The findings indicated that disability (20.4%) increased the risk of falls more than normal ability (15.4%). In addition, the risk of falls in functional decline (22.9%) was higher than that in the normal functional status (13.6%). The proportion of falls in samples with low physical activity (18.3%) was higher than that among those with moderate to high physical activity (15.4%). It was found that the prevalence of falls was 19.4% in single subjects and 14.2% in married subjects. Females (18.3%) experienced a higher rate of falls compared to males (14.8%).

The prevalence of falls was 14.7% among respondents with education and 18.5% among those with no education. Among the samples, 13.3% of Malay and 20.2% of non-Malay ethnicities experienced falls. The results of bivariate analysis showed that the likelihood of falls is not uniform across the elderly with dementia. The findings of chi square tests among respondents showed that the risk of falls significantly associated with ethnicity (*χ* ^2^ = 10.20, *P* = 0.001), disability (*χ* ^2^ = 4.18, *P* = 0.026), the environmental quality (*χ* ^2^ = 11.05, *P* = 0.001), marital status (*χ* ^2^ = 5.75, *P* = 0.010), and functional decline (*χ* ^2^ = 14.44, *P* = 0.001). It was found that the risk of falls was not significantly affected by the environmental facility, physical activity, sex differences, and educational level (*P* > 0.05) (Table 2).

The results of multivariate logistic regression analysis showed the significant effects of ethnicity (*P* = 0.002), functional decline (*P* = 0.019), and the environmental quality (*P* = 0.011) on the risk of falls in older people with dementia. The results indicated that age, disability, physical activity, educational level, marital status, sex differences, and the environmental facility were not significant predictors of risk for having fall in subjects (*P* > 0.05). Although functional decline (OR = 1.67, 95% CI: 1.09–2.57) and non-Malay ethnicity (OR = 1.73, 95% CI: 1.22–2.45) significantly increased the risk of falls, the improved environmental quality (OR = 0.64, 95% CI: 0.45–0.90) reduced the rate of falls in respondents (Table 3).

## 4. Discussion 

Dementia increases the risk of falls. The elderly with dementia experience falls four to five times more than those without cognitive problems [1, 24] which is because of the impairment of judgment, orientation, and visuospatial perception [7]. In addition, the risk of falls in the elderly associates with age related changes in the body systems [25], functional status, physical activity, and environmental conditions [7]. The current study investigated the effects of age, ethnicity, disability, marital status, physical activity, sex differences, educational level, functional status, and the environmental conditions on the risk of falls in the Malaysian elderly with dementia. The results showed that ethnicity, functional status, and the environmental quality significantly predicted the risk of falls. The increased risk of falls probably was due to cognitive impairment, balance problems, and insufficient mobility [26]. Medications, cognitive decline, physical problems, psychological conditions, vision problems, and orthostatic blood pressure [7] can elevate the risk of falls due to mobility deficits and the effect on balance in subjects.

Our findings were in line with the existing reports indicating a direct correlation between functional decline and further risk of falls in the elderly [7, 21, 26]. The results showed that the increased environmental quality decreased the risk of falls in demented elderly. It sounds that an appropriate environmental quality potentially helps respondents to overcome deficits in physical fitness and cognitive abilities posed by dementia and therefore reduces the risk of falls [10, 12]. Surprisingly, physical activity, disability, and the environmental facility had no significant effects on the risk of falls in the Malaysian elderly with dementia. Apparently, the influence of environmental facility on the risk of falls depends on the kind of hazards and functional decline. Furthermore, the risky behaviors of respondents and their attitudes can potentially enhance the risk of falls [11]. Meanwhile, the lack of a uniform scientific definition for potential hazards and a standardized identification as well as diverse instrumental assessments may affect results [27].

The present study confirmed an earlier research [5, 16, 28–30] indicating no significant relationship between physical activity and increased risk of falls. It seems that the effect of physical activity is probably associated with the amount of physical activity and the type of activity [28]. In addition, income, sex differences, marital status, educational level [31], and the environmental quality are possible confounding factors to interact between physical activity and cognition when considering the risk of falls among demented elderly.

Our results showed no correlation between disability and the risk of falls in respondents. Such effect was probably due to the differences in physical, behavioral, environmental, and social factors [13] among subjects. Disability associates with health [10, 13] and age related loss of physical conditions [13]. Therefore, disability accompanies less functions, activities, and participation of demented elderly in dangerous situations and reduces the risk of falls consequently.

Our research confirmed the reported direct correlation between ethnicity and the risk of falls in the elderly [1, 7, 32] which was contrary to some reports [33–35]. Such inconsistency in results is probably due to the differences in predisposition, lifestyle, and cultures [36]. In addition, correlation of age to the risk of falls in earlier reports [1, 9, 37, 38] was found unlikely in our research. Lack of such link in subjects can be explained by the influences of contributing factors interfering with the effects of age on the risk of falls. In this study, marital status, sex differences, and educational level did not predict probability of falls in respondents. While several previous research [1, 7, 39] indicated no correlation between sex differences and the risk of falls, Bueno-Cavanillas and colleagues [40] found a similar pattern to our findings.

Contrary to our research, marital status [1] and educational level [1, 7, 41, 42] have been reported to have impacts on the rate of falls among elderly. Such disparity is probably due to interfering and influencing factors such as income [43], supplement intake [7], social support [44], sleep disorders, and the environmental quality on the risk of falls in the elderly with dementia. As falls can cause much burden and costs in demented elderly and their caregivers [45, 46], further investigations are needed to identify all possible risk factors associated with dementia.

## 5. Conclusions

We concluded that ethnicity, functional status, and the environmental quality significantly predicted the risk of falls in the elderly with dementia. It was found that functional decline and ethnic non-Malay increased the risk of falls. Furthermore, the increased environmental quality reduced the likelihood of falls. Increased knowledge about dementia can enhance capability of controlling physical and psychological damages in the elderly with dementia. As fall is a major health issue in the elderly and dementia, further studies are required to find out the way to prevent or reduce falls in the older individuals with dementia. Such findings help to reduce cost and burden on demented elderly and provide them with a healthy life. There were some factors to limit this study. One of those factors was trouble communicating and collecting accurate data from subjects. Furthermore, the presence of cognitive decline and high prevalence of comorbidities in respondents could interrupt the identification of certain causes of falls.

## Figures and Tables

**Table 1:** Prevalence of falls among 1210 elderly with dementia.

Character	*n*	*n* (%)	95% CI
Falls			
Yes	206	17	15.01–19.24
No	1004	83	80.76–84.99

**Table 2:** Prevalence of falls and associations with sociodemographic factors.

	Whole	*n*	*n* %	95% CI	*χ* ^2^	*P* value
Environment quality						
<5 factors*	280	66	23.6	18.98–28.88	11.05	0.001
≥5 factors	930	140	15.1	12.90–17.49		
Environment facility						
≤3 factors	1052	186	17.7	15.49–20.1	2.45	0.070
>3 factors	158	20	12.7	8.35–18.75		
Disability						
Normal	823	127	15.4	13.12–18.06	4.18	0.026
Disabled	328	67	20.4	16.42–25.12		
Functional test						
<20 s*	559	76	13.6	11.01–16.69	14.44	0.001
≥20 s	389	89	22.9	18.98–27.31		
Physical activity						
Low	672	123	18.3	15.56–21.4	1.75	0.106
Moderate to high	538	83	15.4	12.62–18.73		
Sex differences						
Males	438	65	14.8	11.82–18.47	2.32	0.074
Females	772	141	18.3	15.69–21.14		
Marital status						
Single	665	129	19.4	16.57–22.58	5.75	0.010
Married	543	77	14.2	11.5–17.37		
Ethnicity						
Malays*	550	73	13.3	10.69–16.36	10.20	0.001
Non-Malays	658	133	20.2	17.32–23.45		
Educational level						
No	775	143	18.5	15.88–21.33	2.82	0.054
Yes	430	63	14.7	11.62–18.31		

Significant at the 0.05 level using the chi-square test.

*Reference group.

**Table 3:** Prevalence of falls and associations derived by logistic regression analysis.

	*B*	SE	*P* value	OR	95% CI for OR	
					Lower	Upper
Physical activity	0.070	0.177	0.690	1.07	0.76	1.52
Disability	−0.309	0.183	0.091	0.73	0.51	1.05
Functional test	0.514	0.219	0.019	1.67	1.09	2.57
Environment quality	−0.454	0.179	0.011	0.64	0.45	0.90
Environment facility	−0.172	0.261	0.511	0.84	0.51	1.41
Sex differences	−0.250	0.191	0.190	0.78	0.54	1.13
Educational level	−0.013	0.186	0.944	0.99	0.69	1.42
Marital status	−0.091	0.186	0.626	0.91	0.64	1.31
Ethnicity	0.549	0.177	0.002	1.73	1.22	2.45
Age	0.024	0.012	0.055	1.02	1.00	1.05

Abbreviations: CI, confidence interval; SE, standard error; OR, odds ratio.

Significant at the 0.05 level using the logistic regression analysis, Hosmer-Lemeshow test: *χ* ^2^(8) = 5.22; *P* = 0.734.
